# The circulating form of neprilysin is not a general biomarker for overall survival in treatment-naïve cancer patients

**DOI:** 10.1038/s41598-019-38867-2

**Published:** 2019-02-22

**Authors:** Noemi Pavo, Henrike Arfsten, Anna Cho, Georg Goliasch, Philipp E. Bartko, Raphael Wurm, Claudia Freitag, Heinz Gisslinger, Gabriela Kornek, Guido Strunk, Markus Raderer, Christoph Zielinski, Martin Hülsmann

**Affiliations:** 1 Department of Internal Medicine II, Clinical Division of Cardiology, Medical University of Vienna, Vienna, Austria; 2 Department of Internal Medicine I, Clinical Division of Oncology, Medical University of Vienna, Vienna, Austria; 3 Complexity Research, Vienna, Austria; 4 FH Campus Vienna, Vienna, Austria; 5 Technical University Dortmund, Dortmund, Germany

## Abstract

The transmembrane zink-metalloendopeptidase neprilysin (NEP) is implicated in cardiovascular disease but also tumor biology. The aim of the study was to investigate the relationship of circulating NEP (cNEP) levels with established cardiovascular biomarkers and its effect on overall survival in an unselected cohort of treatment-naïve cancer patients. 555 consecutive cancer patients prior anticancer therapy were enrolled prospectively. NEP levels were determined alongside routine laboratory parameters, established cardiac biomarkers, i.e. NT-proBNP, hsTnT, MR-proANP, MR-proADM, CT-proET-1 and Copeptin, and inflammatory parameters, i.e. CRP, IL-6 and SAA, in venous plasma samples. All-cause mortality was the primary endpoint. cNEP levels of 276 pg/ml (IQR: 0–5981) displayed a weak inverse correlation with age [r = −0.12, p = 0.023] and inflammatory status [r = −0.14, p = 0.007 CRP; r = −0.20, p < 0.001 IL-6 and r = −0.18, p < 0.001 SAA]. cNEP was comparable between different tumor entities and stages and not related to functional parameters of other organ systems as kidney, liver or especially the heart. Moreover, cNEP was not associated with overall survival in the total cohort [adj.HR for ln (cNEP) 1.00, 95% CI: 0.94–1.06, p = 0.887] but in myelodysplatic malignancies [adj.HR for ln (cNEP) 1.27, 95% CI: 1.01–1.61, p = 0.044]. In conclusion, cNEP lacks association with outcome but for myelodysplastic disease. cNEP shows no correlation with established cardiovascular biomarkers related to prognosis, thereby holding a limited potential as a biomarker in cardio-oncology.

## Introduction

Neprilysin inhibition (NEPi) as part of the therapy with angiotensin-receptor neprilysin inhibitor (ARNI) has recently been shown to impressively reduce hospitalization and all-cause mortality in patients with heart failure with reduced ejection fraction (HFrEF)^1^, and now represents standard of care^2^. Consequently, cardiac research has been focusing on revealing the exact mechanisms of action of NEPi. Several reports on concentrations of the circulating form of the enzyme and its activity have been published in the last three years, and circulating NEP concentrations (cNEP) have been discussed controversially as a biomarker for heart failure patients^3,4^. Cardio-oncology is an emerging interdisciplinary field aiming to preserve or stabilize cardiac function in cancer patients receiving anticancer therapy against the background of an aging population accompanied by an increasing burden of both cardiac and malignant disease^5^. Biomarkers may identify patients at risk for long-term cardiotoxicity and currently clinicians rely on the established markers as N-terminal pro B-type natriuretic peptide (NT-proBNP) and high-sensitive TroponinT (hsTnT)^5^. However, several studies have shown elevation of these markers in treatment-naïve cancer patients assumedly as a response to systemic inflammation, making the interpretation of these markers more complex^6^. Molecules implicated in both cardiac and malignant disease could be good candidates for additional characterization of this special patient population.

The zink-metalloendopeptidase or neutral endopeptidase NEP is a member of a class of widely expressed cell surface proteins. NEP is a transmembrane enzyme regulating the physiological action of many peptides as substance P, adrenomedullin, atrial natriuretic peptide, opioids or angiotensins by lowering their extracellular concentration through inactivation by cleavage^7^. Through to its actions on beta-amyloid and vasoactive peptides NEP holds a role in neurological and cardiovascular disorders^8,9^. In HFrEF, the mechanism of NEPi might be based on shifting the homeostasis of vasoactive peptides translating into better clinical outcomes. NEP was originally purified from kidney but is similarly strongly expressed on many other cells and tissues as early B-cells, epithelia of breast, lung, prostate, stomach or colon cells or in the central nervous system. As NEP is a cell surface marker of many stem cells and shows differential expression throughout organ development, it has been proposed as major stem cell regulator protein^7^. NEP seems also to be involved in the function of the immune system. NEP is present on neutrophils and regulates their responsiveness by the degradation of inflammatory peptides^10^. Regarding malignant disease, NEP was formerly described as the tumor-specific antigen in leukemia (common acute lymphoblastic leukemia antigen (CALLA) or CD10), where it is still used to establish diagnosis. However, in later years it became clear, that functions of NEP are far more extensively implicated in oncogenesis and the regulation of tumor microenvironment^7^. In hematopoietic malignancies, NEP overexpression is not restricted to leukemias, but NEP staining is also used for the diagnosis of B-lymphoblastic leukemia or lymphoma cells^11–13^. Alterations in NEP expression have also been reported in solid tumors as colorectal, hepatocellular, lung, cervix or breast cancer and melanoma^14–22^. NEP has been evaluated for its ability to differentiate between primary and secondary tumors of the liver^23^. Intense NEP staining generally seems to indicate a poor prognosis in most solid tumors.

Although NEP is a transmembrane enzyme, a soluble form has been shown to be present in several body fluids as urine, cerebrospinal fluid or plasma, with measurable catalytic activity^24–27^. There is in-vitro evidence that endothelial cells can release NEP in its active form^28^. Although NEP seems to play a key role in tumor biology with potential prognostic information, studies assessing cNEP concentrations in cancer patients are lacking up to date. Moreover, the relationship of cNEP with other established cardiac biomarkers in cancer patients is of interest to characterize the role of NEP in cardio-oncology.

The aim of this study was to investigate whether cNEP levels are specific for distinct cancer types or associated with outcome in an unselected cohort of treatment-naïve cancer patients. Additionally, we aimed to determine the association of cNEP with other established cardiac biomarkers known to reflect cardiac dysfunction and holding prognostic information in this patient population.

## Materials and Methods

### Study population

Between April 2011 and June 2013 we have enrolled consecutive patients with a primary diagnosis of cancer at the Vienna General Hospital, a university-affiliated tertiary care center. Eligible patients presented with suspected or confirmed diagnosis of cancer. Exclusion criteria composed of a history of prior anticancer therapy, clinical signs of infection or if the diagnosis of cancer was not confirmed after the initial work-up. Patients were classified according to tumor entity and tumor stage. Comorbidities as hypertension or diabetes mellitus, traditional risk factors as smoking status and medical therapy were recorded. Patients were followed up for at least 24 months. Written, informed consent was obtained from all study participants. The study protocol complies with the Declaration of Helsinki and was approved by the local ethics committee of the Medical University of Vienna (EK 736/2010).

### Laboratory analysis

Venous blood samples were drawn at first hospital presentation and samples were analyzed according to local laboratory standard procedures. Routinely available laboratory parameters as creatinine, hemoglobin or albumin were measured. Since a former study within this cohort investigated the interrelation of cardiac function and malignant disease^6^, multiple morphologic and functional cardiac markers as hsTnT, NT-proBNP but also mid-regional pro-atrial natriuretic factor (MR-proANP), mid-regional pro-adrenomedullin (MR-proADM), C-terminal endothelin-1 (CT-proET1) and copeptin, the stable, but inactive fragment of the vasopressin (AVP) prohormone, were determined. Also several inflammatory markers, i.e. C-reactive protein (CRP), serum-amyloid A (SAA) and interleukin-6 (IL-6), were measured. Circulating NEP was then additionally determined.

### Assays

Circulating NEP levels were measured in ethylenediaminetetraacetic acid (EDTA) plasma with a specific solid phase sandwich enzyme-linked immunosorbent assay (ELISA) (DY1182, R&D Systems, Minneapolis, USA) according to the manufacturer’s manual. Measurement of samples with an initial dilution of 1:2 yielding values out of the linear range were repeated using higher dilution factors. The analytical parameters for the assay were intra-assay coefficient of variation (CV): <15%, inter-assay CV: 14%, limit of detection: 125 ng/l, and confirmed linearity: 150 to 10000 ng/l. hsTnT and NT-proBNP measurements were performed in heparin plasma using the Elecsys Sytem (Roche Diagnostics, Mannheim, Germany). MR-proANP, MR-proADM, CT-proET-1 and Copeptin were measured in EDTA plasma using specific ELISA (BRAHMS, Hennigsdorf/Berlin, Germany). CRP and SAA levels were determined in EDTA and heparinized plasma by means of particle enhanced immunonephelometry using the BN II System (Siemens Healthcare Diagnostics, Marburg, Germany). Serum IL-6 was detected with a specific enzyme-linked immunosorbent assay (eBioscience, Vienna, Austria).

### Study endpoint

All-cause mortality was chosen as the primary study endpoint. Data were obtained from the Central Office of Civil Registration Austria.

### Statistical analysis

Continuous data were presented as median and IQR and categorical data as counts and percentages. Medians between groups were compared using the Mann-Whitney-U or Kruskal-Wallis test. The Spearman-Rho correlation coefficient was calculated for circulating NEP and other variables. Cox proportional hazard regression analysis was used to evaluate the effect of circulating NEP on all-cause mortality in the total cohort and subgroups of cancer patients. To account for potential confounding effects, multivariate Cox regression analysis was performed adjusting for a clinical confounder model including age, gender and renal function (GFR), and additionally for tumor entity and stage. Results are presented as HRs. For the Cox regression analysis cNEP, NT-proBNP, MR-proANP, MR-proADM, CT-proET-1, and Copeptin were entered in a logarithmic form. Interaction term analysis was performed to determine the influence of metastatic/non-metastatic disease state on the association of cNEP with overall survival. To assess the association of cNEP levels with the primary endpoint graphically, the total population and subgroups with most common malignant entities were divided into tertiles and overall survival for 1200 days was presented as Kaplan Meier curves. Groups were compared by the means of the log-rank-test. For all tests two-sided p-values lower 0.05 were considered to indicate statistical significance. The analyses were carried out using the SPSS 22.0 software (IBM Corp, New York, NY, USA).

## Results

### Baseline characteristics

A total of 555 consecutive patients were included in the study. The detailed baseline characteristics of our study population, as already presented in a related manuscript, are displayed in Table 1, a complete description of tumor entities is presented in Supplemental Table 1. Median age was 62 (IQR 52–71) and 41% of the patients were male. 33% of patients presented with a tumor stage 4. CRP was determined with a median of 0 mg/dl (IQR 0–1), SAA with 8 µg/ml (IQR 4–16) and IL-6 with 2 pg/ml (IQR 2–3) for the total cohort. Median cNEP values of the total cohort were 276 pg/ml (IQR 0–5981), showing a wide range. cNEP was under the detection limit in 161 (29%) of the samples, whereas 22 (4%) samples displayed highest concentrations entered as 200000 pg/ml.

**Table 1:** Baseline characteristics of treatment-naïve patients diagnosed with cancer (n = 555).

	Treatment-naïve cancer patients (n = 555)
Age, years (IQR)	62 (52–71)
Male gender, n (%)	227 (41%)
BMI, kg/m^2^ (IQR)	25.0 (22.6–28.4)
Comorbidities	
Known CAD, n (%)	28 (5%)
Heart Failure, n (%)	38 (7%)
Diabetes mellitus, n (%)	43 (8%)
Arterial Hypertension, n (%)	250 (45%)
CKD, n (%)	31 (6%)
COPD, n (%)	113 (20%)
Cancer disease stage*	
Stage 1, n (%)	96 (17%)
Stage 2, n (%)	50 (9%)
Stage 3, n (%)	108 (19%)
Stage 4, n (%)	183 (33%)
Cardiac biomarkers	
hsTnT, ng/ml (IQR)	0.006 (0.003–0.010)
NT-proBNP pg/ml (IQR)	128 (64–279)
MR-pro-ANP, pmol/l (IQR)	66 (47–107)
MR-proADM, nmol/l (IQR)	0.56 (0.44–0.72)
CT-proET-1, pmol/l (IQR)	52.5 (40.3–68.1)
Copeptin, pmol/l (IQR)	4.60 (2.90–8.28)
Laboratory parameters	
GFR, mL/min/1.73 m^2^ (IQR)	74.5 (63.7–86.0)
BUN, mg/dl (IQR)	15 (12–19)
BChE, kU/l (IQR)	7.31 (6.10–8.40)
AST (GOT), U/l (IQR)	24 (19–31)
ALT (GPT), U/l (IQR)	22 (16–32)
GGT, U/l (IQR)	32 (21–63)
Bilirubin, mg/dl (IQR)	0.58 (0.44–0.79)
Albumin, g/l (IQR)	43.0 (40.0–45.4)
CRP, mg/dl (IQR)	0 (0–1)
SAA, µg/ml (IQR)	8 (4–26)
IL-6, pg/ml (IQR)	2 (2–3)

Continuous variables are given as medians and inter-quartile ranges (IQR). Counts are given as numbers and percentages.

IQR – interquartile range; BMI – body mass index, CAD – coronary artery disease; CKD – chronic kidney disease; COPD – chronic obstructive pulmonary disease; GFR – glomerular filtration rate; BUN – blood urea nitrogen; BChE – butyryl-cholinesterase, AST – aspartate transaminase, ALT – alanine transaminase, GGT – gamma glutamyltransferase; CRP - C-reactive protein; SAA - Serum Amyloid A; IL-6 – interleukin 6.

*Tumor stage was assessed by the respective treating oncologist and was indicated for all patients excluding those with myeloproliferative neoplasias.

### Association of circulating neprilysin with baseline demographic parameters, tumor entity and stage

**Figure 1:**
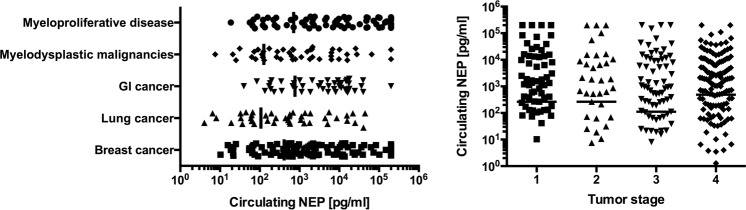
Circulating NEP levels according to tumor entities and disease stage in a treatment-naïve unselected cohort of cancer patients. cNEP levels are represented as individual datapoints, the median is indicated. There were no significant differences in cNEP levels between different tumor entities or between different disease stages. Due to the logartithmic scale cNEP values of 0 cannot be displayed (129 samples).

cNEP showed a weak inverse correlation with age [r = −0.12, p = 0.023] however did not correlate with other baseline demographic parameters as BMI [r = 0.00, p = 0.989], systolic blood pressure [r = 0.01, p = 0.789] or heart rate [r = 0.05, p = 0.349]. Moreover, cNEP levels of men and women were comparable [263 pg/ml (IQR 0–7368) vs 298 pg/ml (IQR 0–5052), p = 0.890]. cNEP concentrations of distinct tumor entities and disease stages are shown in Fig. 1. cNEP levels were comparable between tumor entities and stages. Notably, there was no difference in cNEP levels between disease stages according to the subgroups of the most common malignancies [Supplementary Fig. 1A, p = ns for all] or between metastatic disease, i.e. stage 4, compared to non-metastatic disease, i.e. stages 1–3, neither in the total cohort [198 ng/l (IQR 0–4939) vs 489 ng/l (IQR 0–4260), p = 0.206] nor in the subgroups [Supplementary Fig. 1B, p = ns for all]. Also there were no differences between patients with undetectable cNEP levels compared to patients with measurable cNEP with regards to age, kidney function or more importantly cancer type or disease stage.

### Correlation of circulating NEP with the cardiac and inflammatory biomarkers

There was no significant correlation of cNEP levels with the cardiac biomarkers [r = −0.05, p = 0.367 for NT-proBNP; r = −0.10, p = 0.075 for hsTnT; r = −0.03, r = −0.02, p = 0.664 for MR-proANP; r = −0.05, p = 0.387 for MR-proADM; r = 0.07, p = 0.168 for CT-proET1 and r = −0.01, p = 0.864 for Copeptin]. A weak, but significant inverse correlation was observable for cNEP and the inflammatory markers [r = −0.14, p = 0.007 for CRP, r = −0.18, p < 0.001 for SAA and r = −0.20, p < 0.001 for IL-6].

### Routine laboratory parameters according to circulating NEP tertiles

Table 2 shows the comparison between routine laboratory parameters according to cNEP tertiles. There was no significant difference or trend neither in kidney functional parameters glomerular filtration rate (GFR), creatinine and urea, nor hemoglobin or liver parameters as bilirubin, gamma-glutamyltransferase (GGT), aspartate transaminase (AST), alanine transaminase (ALT), butyryl-cholinesterase (BChE) and albumin for the different cNEP tertiles.

**Table 2:** Circulating NEP levels and laboratory parameters according to tertiles in unselected treatment-naïve cancer patients (n = 555).

	1. Tertile	2. Tertile	3. Tertile	p-value
Circulating NEP, pg/ml (IQR)	0 (0–0)	276 (90–768)	13651 (5981–50947)	—
GFR, mL/min/1.73 m^2^ (IQR)	73.64 (63.98–86.45)	74.47 (61.67–86.79)	74.60 (63.71–84.69)	0.775
Creatinine, mg/dl (IQR)	0.87 (9.76–1.00)	0.89 (0.76–1.01)	0.88 (0.79–1.05)	0.334
BUN, mg/dl (IQR)	14 (12–19)	16 (12–20)	16 (12–19)	0.114
Hemoglobin, mg/dl (IQR)	13.4 (12.0–14.4)	13.3 (11.9–14.3)	13.1 (11.9–14.3)	0.757
Bilirubin, mg/dl (IQR)	0.57 (0.41–0.77)	0.59 (0.46–0.77)	0.60 (0.44–0.83)	0.375
GGT, U/l (IQR)	30 (19–53)*^,§^	38 (23–73)*	32 (23–63)^§^	**0**.**007**
AST (GOT), U/l (IQR)	23 (19–29)	24 (18–32)	25 (20–34)	0.166
ALT (GPT), U/l (IQR)	22 (17–29)	21 (15–35)	24 (18–34)	0.128
BChE, kU/l (IQR)	7.34 (5.89–8.35)	7.30 (5.92–8.35)	7.31 (6.37–8.72)	0.477
Albumin, g/l (IQR)	43.2 (39.6–45.7)	43.0 (39.4–45.3)	43.0 (40.6–45.4)	0.928

Fonts in bold indicate statistical significance (p < 0.05).

^*^For p < 0.05 between the 1. and 2. tertile; ^$^for p < 0.05 between the 1. and the 3. tertile.

### Survival analysis

186 (34%) patients of the total cohort died during a median follow-up of 27 (IQR 18–32) months. Table 3 shows the association of circulating NEP with outcome for the total cohort as well as the most common tumor entity subgroups. cNEP was not associated with all-cause mortality neither in the univariate analysis [crude HR for ln (cNEP) 0.97, 95% CI: 0.92–1.02, p = 0.253] nor after adjustment for age, gender, kidney function, tumor entity and tumor stage [adjusted HR for ln (cNEP) 1.00, 95% CI: 0.94–1.06, p = 0.887]. There was no significant interaction of metastatic/non-metastatic disease state with regards to the predictive value of cNEP [p = 0.434 for interaction]. No association with outcome could be shown for the solid tumor entities as breast cancer, lung cancer or gastrointestinal cancers or myeloproliferative disease, however cNEP was a significant risk factor for adverse outcome in myelodysplastic disease [adjusted HR for ln (cNEP) 1.27, 95% CI: 1.01–1.61, p = 0.044].

**Table 3:** Association of circulating NEP levels with all-cause mortality in unselected treatment-naïve cancer patients according to tumor site (n = 555).

	Crude HR	p-value	Adj. HR	p-value
Total cohort (n = 555)	0.97 (0.92–1.02)	0.253	1.00 (0.94–1.06)^*^	0.887
Breast cancer (n = 146)	0.97 (0.82–1.14)	0.671	0.99 (0.84–1.16)^#^	0.891
Lung cancer (n = 61)	0.97 (0.85–1.11)	0.666	0.97 (0.85–1.11)^#^	0.663
Gastrointestinal cancer (n = 67)	0.96 (0.80–1.14)	0.610	0.97 (0.81–1.17)^#^	0.776
Myelodysplastic neoplasia (n = 68)	1.27 (1.01–1.60)	**0**.**041**	1.27 (1.01–1.61)^#^	**0**.**044**
Myeloproliferative disease (n = 99)	0.97 (0.73–1.27)	0.810	0.99 (0.75–1.31)^#^	0.938

Fonts in bold indicate statistical significance (p < 0.05).

*Adjusted for age, gender, kiney function (GFR), tumor entity and stage.

^#^Adjusted for age and kidney function (GFR).

### Kaplan Meier curves

**Figure 2:**
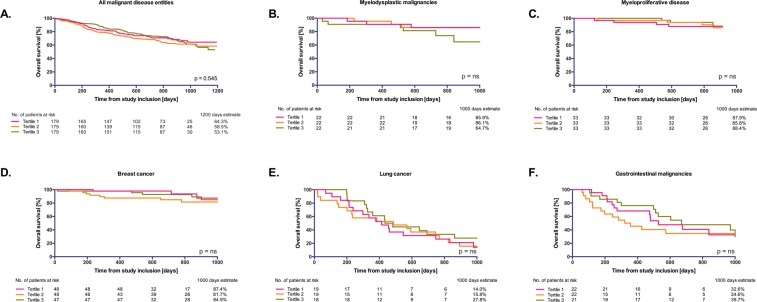
Association of circulating NEP with all-cause mortality. Overall survival rates for (**A**) the total cohort of treatment-naïve cancer patients (n = 555) according to tertiles of circulating NEP (p = 0.545 between all groups, log-rank test) and stratified to the most common malignant disease entities, i.e. (**B**) myelodysplastic disease, (**C**) myeloproliferative disease, (**D**) breast cancer, (**E**) lung cancer and (**F**) gastrointestinal malignancies.

Kaplan Meier curves and log-rank analysis for the total cohort and subgroups of the most common tumor entities, i.e. myelodysplastic and myeloproliferative disease, breast cancer, lung cancer and gastrointestinal malignancies, are shown in Fig. 2. For the total cohort, the 12 and 24 month estimates were 82.7% and 70.7% in the lower, 79.3% and 68.2% in the mid and 86.0% and 73.3% in the upper tertile, confirming the lack of discriminatory power of cNEP on overall survival for treatment-naïve cancer patients (p = 0.545 between all groups). Moreover, no differences in overall survival could be observed for the respective subgroups regarding cNEP tertiles (p = ns for all). Overall survival was better for patients with myelodysplastic or myeloproliferative malignancies and breast cancer than for gastrointestinal and lung cancer.

## Discussion

This is the first report investigating the suitability of the circulating form of the ubiquitous zink-metalloendopeptidase neprilysin as a prognostic biomarker in cancer patients. cNEP concentrations show a wide range in plasma of cancer patients displaying a weak inverse correlation with age and inflammatory state, but not with gender or BMI. Contrary to our hypothesis, cNEP holds no ability to predict outcome neither in the total cohort of unselected cancer patients, nor in the subgroups of solid tumors or myeloproliferative disease. Myelodysplastic disease is the only entity where prognosis of patients is associated with cNEP levels, whereas here cNEP represents a risk factor for all-cause mortality. Especially, no relationship could be established with functional parameters of different organ systems including the heart, characterized by a set of well-established cardiac biomarkers.

Generally, there are only several reports studying circulating NEP concentrations, and none of them has been conducted in cancer patients. In healthy subjects, cNEP levels were determined with 0.155 nmol/l (IQR 0.048–0.310) for subjects with a BMI <25 kg/m^2^ – corresponding to approximately 133 pg/ml (IQR 41–265) considering the molecular weight of the total protein - and showed a positive correlation with BMI and insulin resistence^29^. In heart failure patients cNEP levels were indicated with 642 pg/ml (IQR 385–1219) and 2862 pg/ml (IQR 2068–3827), whereas different assays were used in different cohorts^3,4^. A study investigating dyspneic patients revealed higher cNEP levels for chronic heart failure patients compared to individuals without heart failure [352 pg/ml vs 256 pg/ml]^30^, whereas a more recent report highlighted the contribution of the myocardium to cNEP levels in advanced heart failure^31^. Regarding its predictive value, cNEP levels in CKD patients were not associated with future hospitalizations for heart failure^32^. In these reports cNEP either did not correlate or showed a weak but direct correlation with age^3,4^. Our cohort of cancer patients displayed a wide range of cNEP levels with 276 pg/ml (IQR 0–5981), a weak inverse correlation with age and no correlation with BMI. This was the first study to use the ELISA kit from R&D Systems for plasma measurements. This kit has only been used previously for tissue homogenates of colorectal cancer cells or brain tissue^18,33^. The fact, that the analytical process of measuring cNEP is puzzling, has recently been shown in a manuscript, where different commercially available immunoassays simply yielded an alarming lack of correlation^34^. The reason for this may lie in multiple factors including lack of reference measurements and materials, different antibody clonalities combined with the unknown sequence of cNEP and possible posttranslational modifications at an early stage of scientific interest. The R&D kit has not been tested in comparison.

Because increased NEP expression is mostly characteristic for more advanced malignant disease, we have hypothesized, that circulating NEP would be a risk factor for adverse outcome in various type of treatment-naïve cancer patients. Indeed, the role of NEP as a marker of hematopoietic malignancies has been early recognized. CD10 was discovered in acute lymphoblastic leukemia as a cancer specific antigen but also 80% of patients with non-T ALL show enhanced CD10 expression^11,35^. Enhanced CD10 expression was further shown for different B-cell lymphomas and rarely also for T-cell lymphomas^12^. CD10 is clinically used for differential diagnosis of B-lineage lymphoid neoplasms^13^. However soon it became evident, that due to its widely expression and multiple biologic actions, deregulated NEP expression is not specific for hematopoietic malignancies. High NEP expression has found to be associated with poor prognosis in non-small cell lung cancer and tumoral NEP was an independent predictor of recurrence of stage I lung adenocarcinomas^14,15^. In breast cancer the role of NEP seems to be more complex. NEP overexpression was related to improved disease-free survival and a decreased breast cancer cell invasion *in-vitro*^16,17^. However, NEP expression of stromal cells again seem to correlate with poor prognosis and estrogen receptor negativity^36^. Increased NEP expression has equally been described in colorectal cancer and hepatocellular carcinoma and is particularly associated with liver metastasis in colorectal cancer^18,23,37^. Consequently, an involvement of deregulated NEP expression has been described for several other tumor entities, as melanoma or carcinomas originating from the kidney, cervix, bladder or prostate, with partly conflicting results^19–22^.

Contrary to our assumption cNEP seems not to be associated with outcome in cancer patients generally, and neither in common solid tumor entities as breast cancer, lung cancer or malignant disease of the gastrointestinal tissue, nor in myeloproliferative disease. However, we have found that increased plasma NEP is associated with adverse outcome in myelodysplastic disease including various types of lymphomas and leukemias. The latter finding appears reasonable taking into account that NEP was first discovered and is probably most thoroughly reviewed in myelodysplastic disease. NEP overexpression on malignant hematopoietic cells released into circulation might contribute to the higher levels of detectable NEP in plasma. We can only speculate about the reason for a lacking association of cNEP and prognosis in solid tumors despite higher expression of NEP on malignant tissue. Either NEP on malignant cells does not contribute to detectable NEP levels in plasma at all or its impact is insignificant. Monitoring of cNEP levels across different disease states and during therapy could provide further significant insights into the usefulness of cNEP as a biomarker in malignant disease.

The circulating form of NEP is poorly understood - it is not even clear whether NEP in body fluids represents a released form of the mature enzyme or if it’s a shed fragment of the protein originating from various tissues or blood cells. However, a retained catalytic activity of NEP has been shown in plasma as well as cerebrospinal fluid^38^, which could be a hint for a significant physiological role of this soluble form. In patients with heart failure with reduced ejection fraction higher cNEP concentrations seem to be linked to increased cardiovascular death^4^. However, also here no data exist on the relationship between tissue specific expression or activity and circulating NEP concentrations or activity. From a cardiooncologic point of view it is remarkable, that cNEP does not correlate to any of the well-established cardiac biomarkers as NT-proBNP, hsTnT, MR-proANP, CT-proET-1, MR-proADM or Copeptin. Since the introduction of NEPi into heart failure therapy, there is a huge investigational effort trying to unveil the exact mechanisms lying beyond the convincing clinical data^1,2^. We have previously shown that all of the cardiac biomarkers mentioned above are highly significantly associated with prognosis in this cohort^6^. Based on the literature above suggesting cNEP as a biomarker in heart failure, we would have assumed a correlation of cNEP with the established biomarkers in this cancer patient cohort.

Although cNEP could not be related to outcome, there was a consequent modest association with the inflammatory markers CRP, SAA and IL-6. NEP is expressed on neutrophils and B-cells and thereby seems to regulate immune response^10^. Transgenic CD10−/− knockout mice display a 10-fold sensitivity to endotoxin stimulation and die much faster than wildtype animals^10^. A small FACS study described CD10 expression as a marker of neutrophil function capacity with decreased levels in septic patients and a dynamic response to endotoxin *in vitro*^39^, so that consequently, CD10 expression was suggested as a possible cell surface biomarker of sepsis^40^. Again, if surface expression of NEP on leukocytes contributes to the levels of detectable plasma NEP, an inverse correlation of cNEP to inflammatory status would be in line with the literature data mentioned above.

## Conclusions

Cancer patients show a wide range of circulating NEP levels. Although NEP is implicated in oncogenesis and tumor progression on the tissue level with mostly overexpression of NEP in more advanced malignant stages, there is no general association of the circulating form of NEP with survival. In different leukemias and lymphomas, the regular NEP expression on leukocytes is well-known to be altered, and in these entities plasma NEP seems to be related to adverse outcome. Regarding cardiac involvement circulating NEP does not seem to be a good biomarker for subclinical cardiac dysfunction in cancer.

## Limitations

One potential limitation of this study is the unselective nature of patient enrollment including various types of cancer. Nevertheless, we intended to investigate biomarkers as a general phenomenon in cancer, without focusing on distinct tumor entities. Further studies might reveal more differences between various types of cancer. Laboratory measurements have been performed only at a single time point prior to initiation of anticancer therapy and studies with serial measurements throughout disease progression might provide additional insights. While our endpoint is all-cause mortality, precise information about the percentage of cancer-related death or other endpoints, e.g. progression-free survival or quality of life, would certainly be of important clinical interest. Finally, there is little known about the circulating form of NEP and there appears to be a low conformity between plasma measurements with commercial immunoassays, whereas this is the first study using the above mentioned kit in human plasma samples. Whether the relatively high number of patients with non-detectable cNEP is a cancer specific finding or depends on the assay used has to be investigated in future studies.

## Supplementary information

Supplementary Material

## Data Availability

The datasets generated during and/or analysed during the current study are available from the corresponding author on reasonable request.
