# Supplementary material for: The circulating form of neprilysin is not a general biomarker for overall survival in treatment-naïve cancer patients
Source: Sci Rep. 2019 Feb 22;9:2554. doi: 10.1038/s41598-019-38867-2 (PMC6385211; doi:10.1038/s41598-019-38867-2)
Supplement: Supplementary file 1 — Supplementary Material [file 41598_2019_38867_MOESM1_ESM.docx]

**The circulating form of neprilysin is not a general biomarker for overall survival in treatment-naïve cancer patients**

Noemi Pavo MD^*^, Henrike Arfsten MD^*^, Anna Cho^*^, Georg Goliasch MD^*^, Philipp E. Bartko MD^*^, Raphael Wurm MD^*^, Claudia Freitag^*^, Heinz Gisslinger MD^‡^, Gabriela Kornek MD^‡^, Guido Strunk MSc PhD^§^, Markus Raderer MD^‡^, Christoph Zielinski MD^‡^, Martin Hülsmann MD^*^

* Department of Internal Medicine II, Clinical Division of Cardiology, Medical University of Vienna, Vienna, Austria

^‡^ Department of Internal Medicine I, Clinical Division of Oncology, Medical University of Vienna, Vienna, Austria

^§^ Complexity Research, Vienna, Austria; FH Campus Vienna, Vienna, Austria and Technical University Dortmund, Dortmund, Germany

^*^ **Address for correspondence:**

Martin Hülsmann MD

Department of Cardiology, Medical University of Vienna, Austria

Währinger Gürtel 18-20.

1090 Vienna, Austria

Tel.: +43-1-40400-46140

Fax: +43-1-40400-42160

E-mail: martin.huelsmann@meduniwien.ac.at

**SUPPLEMENTARY FIGURE**

**Supplementary Figure 1. Circulating NEP levels according to disease stage in a treatment-naïve unselected cohort of cancer patients.** cNEP levels are represented as Tukey boxplots. There were no significant differences in cNEP levels between A. tumor stages or B. non-metastatic vs. metastatic disease according to tumor entity. Due to the logartithmic scale cNEP values of 0 cannot be displayed (129 samples).

**
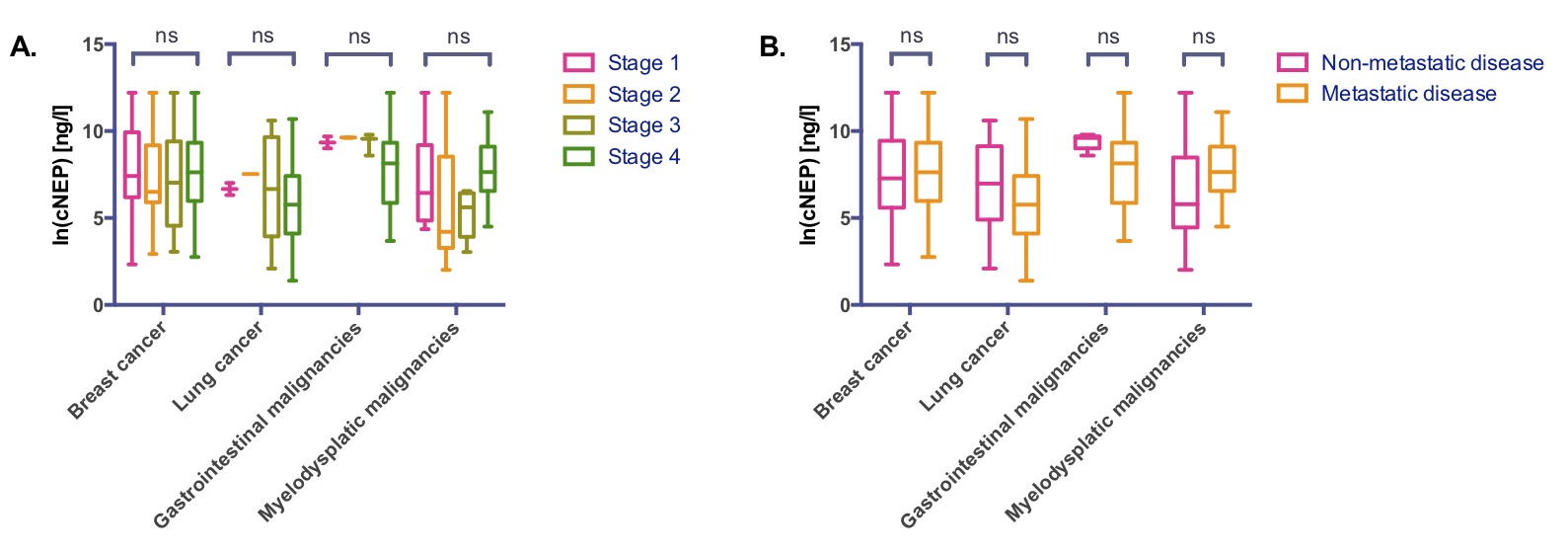
**

**SUPPLEMENTARY TABLE**

**Supplementary Table 1.** Tumor entities of treatment-naïve cancer patients (n=555). Counts are given as numbers and percentages.

|  | **Cancer patients (n=555)** |
| --- | --- |
| **Tumor entity** |  |
| Lung cancer, n (%) | 61 (11.0%) |
| Breast cancer, n (%) | 146 (26.3%) |
| Brain tumor, n (%) | 23 (4.1%) |
| ENT-tumor, n (%) | 33 (5.9%) |
| Gastrointestinal tumors, n (%) | 67 (12.1%) |
| Myelodyplastic malignancies, n (%) | 68 (12.3%) |
| Myeloproliferative neoplasias, n (%) | 99 (17.8%) |
| Esophageal cancer, n (%) | 11 (2.0%) |
| Testicular cancer, n (%) | 2 (0.4%) |
| Neuroendocrine tumor, n (%) | 11 (2.0%) |
| Sarcoma, n (%) | 9 (1.6%) |
| Mesothelioma, n (%) | 3 (0.6%) |
| Prostate cancer, n (%) | 2 (0.4%) |
| Renal cell carcinoma, n (%) | 4 (0.7%) |
| Thymoma, n (%) | 1 (0.2%) |
| Skin cancer, n (%) | 2 (0.4%) |
| Urogenital tumors, n (%) | 2 (0.4%) |
| Oral cancer, n (%) | 1 (0.2%) |
| Other, n (%) | 10 (1.8%) |

ENT – ear, nose, throat; Myelodysplastic malignancies – haematological malignancies with abnormal differentiation of myeloid or lymphoid cell lines (e.g. AML, ALL, lymphomas, multiple myeloma); Myeloproliferative neoplasias – haematological neoplasias with normal cell differentiation (e.g. essential thrombocytosis, polycythemia vera, myelofibrosis).
